# Clinical Profile and Magnetic Resonance Angiographic Findings Among Patients With Cerebrovascular Stroke

**DOI:** 10.7759/cureus.85739

**Published:** 2025-06-10

**Authors:** Cassandra Elaine Griffiths, Madhu A Yadav, Anju Susan Alexander, Gorthi Nikitha Chowdary, Dhanush Shetty, Ishani Pandya, Harikrishnan Sathish Kumar, Prasanna Ratilal Patel

**Affiliations:** 1 General Medicine, Mysore Medical College and Research Institute, Mysore, IND; 2 Anaesthesia and Critical Care, Sri Padmavathi Children's Heart Centre, Tirupati, IND; 3 General Medicine, Christian Medical College Vellore, Vellore, IND; 4 General Medicine, M V Jayaraman Medical College and Research Hospital, Bengaluru, IND; 5 Emergency Medicine, Jagadguru Jayadeva Murugarajendra Medical College, Davanagere, IND; 6 General Medicine, Grodno State Medical University, Grodno, IND; 7 General Medicine, Saveetha Medical College and Hospital, Chennai, IND; 8 General Medicine, Gujarat Medical Education and Research Society (GMERS) Medical College and Hospital, Valsad, IND

**Keywords:** hemorrhagic, ischemic, mr angiography, mri, stroke

## Abstract

Background

Stroke diagnosis is simplified when magnetic resonance imaging (MRI) findings correlate with the clinical features. The present study evaluated the clinical profile in cerebrovascular stroke patients. In addition, the MR angiographic profile of cerebrovascular stroke was assessed, and the clinical and MR angiographic findings were compared in ischemic and hemorrhagic stroke.

Methodology

This was a hospital-based cross-sectional study conducted among 50 stroke patients, including patients clinically diagnosed with stroke, ischemic, and hemorrhagic, presenting within 48-72 h of the onset of symptoms.

Results

In this study, the prevalence of ischemic stroke and hemorrhagic stroke was 40 (80.0%) and 10 (20.0%), respectively. The incidence of seizures (p = 0.007) and headache (p = 0.000) was higher in hemorrhagic stroke when compared to ischemic stroke. The mean fibrinogen level was higher in ischemic stroke when compared to hemorrhagic stroke (441.5 ± 152.04 vs. 308.5 ± 107.3 mg/dL; p = 0.012). The MR angiographic profile was normal in all 10 cases of hemorrhagic stroke; meanwhile, in ischemic stroke, only five (12.5%) had normal findings (p < 0.001). MR angiography (MRA) findings indicated that among ischemic stroke patients, the majority of cases involved the middle cerebral artery (MCA) with nine cases (22.5%), followed by the posterior cerebral artery (PCA) with four cases (10%), and both the internal carotid artery (ICA) and anterior cerebral artery (ACA) with two cases (5%) each.

Conclusion

In this study, ischemic stroke was more prevalent than hemorrhagic stroke. MRA proved valuable in differentiating stroke subtypes and identifying involved vessels in ischemic cases.

## Introduction

A stroke is characterized as a cluster of quickly emerging clinical symptoms indicating focal disruption of brain function that lasts more than 24 h or results in death with no evident cause other than vascular origin. One of the leading causes of death and disability is cerebral ischemic stroke [1]. In India, the incidence rate of stroke across all age groups has exhibited a general upward trend, rising from 76 per 100,000 individuals in 1990 to 88 per 100,000 individuals in 2021. The incidence rate within the 15-49 age group exhibited relative stability, with minor fluctuations observed, consistently remaining within the mid-30s to mid-40s range over the specified period. [2]. There are several forms of stroke, including various dural sinus/cerebral vein obstructions (1%), non-traumatic subarachnoid hemorrhage (5%), primary intracranial hemorrhage (15%), and cerebral infarction (80%). The ischemic stroke is the most prevalent stroke pattern, accounting for 80-90% of all strokes [3]. Ischemic stroke, which results from arterial occlusion, accounts for approximately 80-85% of stroke cases, whereas hemorrhagic stroke occurs due to the rupture of cerebral blood vessels. The clinical manifestations are contingent upon the specific vascular territory affected and may encompass motor weakness, sensory disturbances, speech impairments, altered levels of consciousness, and visual deficits [4].

Stroke imaging assesses the parenchyma, perfusion, penumbra, and pipes (intracranial and extracranial circulation) [5]. This method helps identify intravascular thrombi, distinguish salvageable tissue from infarcted tissue, detect cerebral hemorrhage, choose the best course of treatment, and forecast the clinical prognosis [6]. The most common stroke evaluation approach is computer tomography. However, when it comes to identifying early infarction, MRI is more sensitive and specific than traditional CT. MRIs provide extensive information for early stroke management [7]. MRI shows better detail of soft tissues than CT, which helps find acute ischemic stroke lesions earlier and more accurately, especially right after the stroke happens. Additionally, MRI can better differentiate stroke subtypes and identify smaller infarcts, improving diagnostic accuracy and guiding treatment decisions [7].

A standard stroke magnetic resonance imaging (MRI) protocol includes magnetic resonance angiography (MRA), magnetic resonance spectroscopy (MRS), fluid-attenuated inversion recovery (FLAIR), diffusion-weighted imaging (DWI), apparent diffusion coefficient (ADC), T1-weighted imaging (T1W), and T2-weighted imaging (T2W) [6,7]. The sensitivity of gradient echo (GRE) MR sequences in identifying hemorrhage is high. MRA can evaluate the condition of the neck and cerebral arteries. MRS can assess the state of different metabolites [6]. The extent of potentially recoverable brain tissue, the location of artery occlusion, and the exclusion of ischemic stroke differential diagnoses are all accurately determined by this method [8]. Correlating the MRI results with clinical aspects makes diagnosing easier [6].

This study was conducted with the following objectives: (1) to study the clinical profile in cerebrovascular stroke and the MR angiographic profile of cerebrovascular stroke and (2) to compare clinical and MR angiographic findings in ischemic and hemorrhagic stroke.

## Materials and methods

This hospital-based cross-sectional study was conducted among 50 stroke patients admitted to the Department of General Medicine, Gujarat Medical Education and Research Society (GMERS) Medical College and Hospital, Valsad, India, from March 2024 to March 2025. Ethical approval was obtained from the institutional ethics committee with protocol number GMERS/MCV/IHEC/154/24. Informed consent was obtained from the patient/legal guardian. Confidentiality of the data was ensured.

Inclusion criteria

Patients clinically diagnosed with stroke, both ischemic and hemorrhagic, presenting within 48-72 h of the onset of symptoms and of age >18 years.

Exclusion criteria

Patients of age <18 years; patients with other CNS diseases (e.g., dementia, muscular dystrophy, myasthenia gravis, demyelinating diseases, and infections); patients who have cochlear implants, clips or brain aneurysms, metallic coils in blood vessels, all cardiac defibrillators, and pacemakers; patients presenting after 72 h of the onset of symptoms; patients who have received earlier treatment with anticoagulants; and patients suffering from an acute infectious etiology will be excluded from the study.

Data collection

Data was collected using a structured proforma. Once we obtained informed consent from the patients, we examined them to assess their clinical status upon admission. After a thorough history, general examination, and CNS examination, the patient’s blood panel was sent, and an MRI of the brain with MRA was performed. The MRI sequences included DWI, FLAIR, GRE, T1W, and T2W sequences. MRA was conducted using a time-of-flight (TOF) sequence. The findings were entered in the proforma. Patients were managed with ABC support (airway, breathing, and circulation), antiplatelets, physiotherapy, and treatment of the underlying cause. Patients were monitored closely, and their prognosis was assessed. 

Data analysis

Data was checked for completeness and consistency and analyzed using IBM SPSS Statistics software. Descriptive statistics, percentages, and mean were calculated. Inferential analysis was done using the chi-square test for categorical data and the unpaired t-test for continuous data. A p-value of less than 0.05 was taken as statistically significant.

## Results

**Figure 1:**
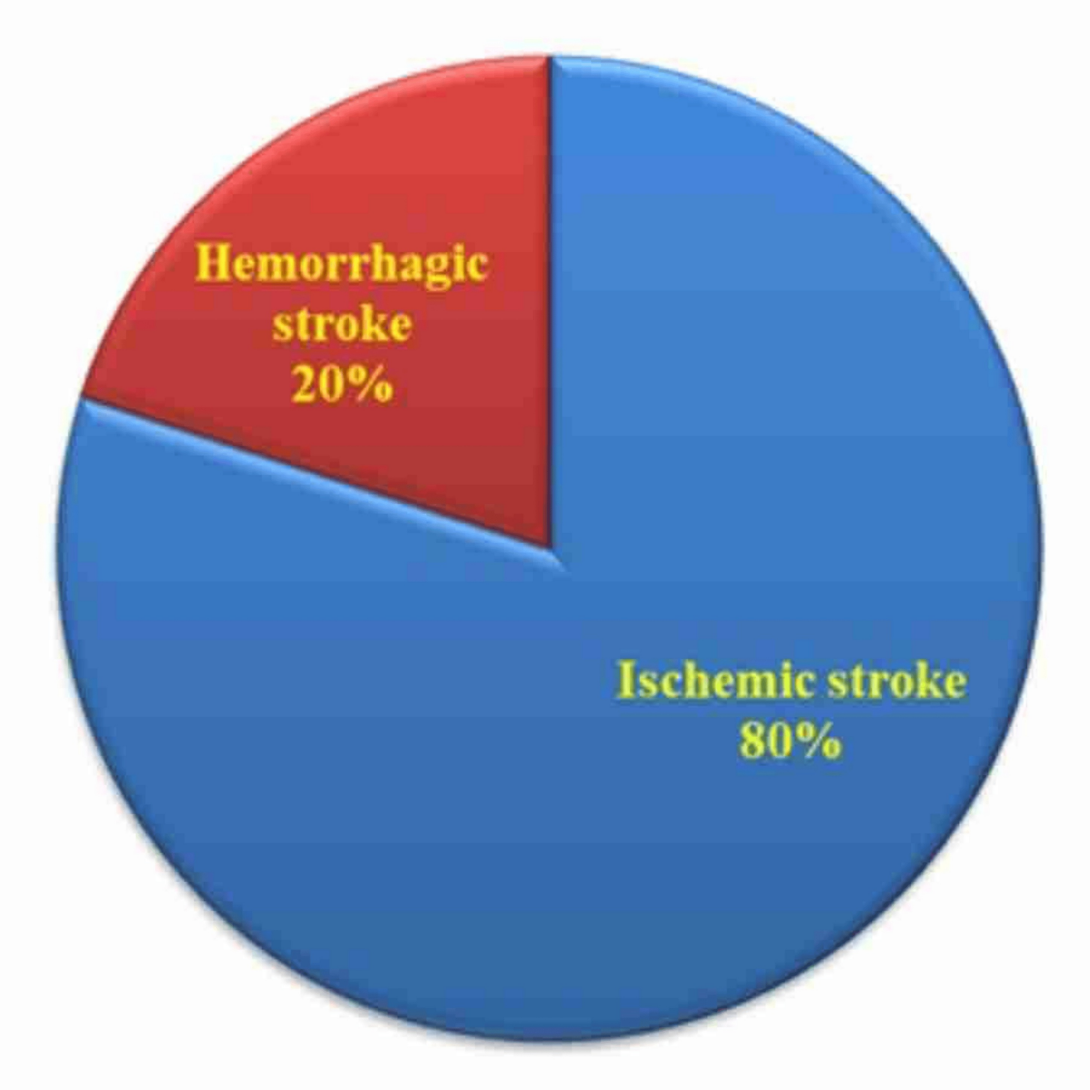
Type of stroke distribution (N = 50)

In this study, out of 50 stroke cases, 40 (80%) were ischemic stroke and 10 (20%) were hemorrhagic stroke. The results are shown in Figure 1.

The prevalence of stroke was higher among males (60%) and those more than 60 years of age (38%). There was no significant difference in age (p = 0.386) and gender distribution (p = 0.470) between the patients with ischemic stroke and hemorrhagic stroke (Table 1).

**Table 1:** Comparison of the demographic characteristics among the ischemic and hemorrhagic stroke patients The data is shown as frequency (N) and percentage (%). NS, non-significant (chi-square test)

Parameters	Ischemic stroke (n = 40), N (%)	Hemorrhagic stroke (n = 10), N (%)	Total, N (%)	Chi-square value	p-value
Age (years)					
18-40	7 (17.5%)	1 (10%)	8 (16%)	3.076	0.38^NS^
41-50	9 (22.5%)	5 (50%)	14 (28%)		
51-60	7 (17.5%)	1 (10%)	8 (18%)		
> 60	17 (42.5%)	3 (30%)	20 (38%)		
Gender					
Male	25 (62.5%)	5 (50%)	30 (60%)	0.522	0.47^NS^
Female	15 (37.5%)	5 (50%)	20 (40%)		

The most common presenting symptom was hemiplegia (82%), followed by speech disorder (68.0%), altered sensorium (56.0%), and facial palsy (44.0%). The remaining symptoms were found as facial palsy (n = 22, 44%), seizure (n = 17, 34%), involuntary urination and defecation (n = 11, 22%), headache (n = 10, 20%), and vomiting (n = 10, 20%). However, the incidence of hemiplegia was higher in patients with hemorrhagic stroke compared to those with ischemic stroke (85% vs. 70%). Seizures (p = 0.007) and headache (p = 0.000) were significantly higher among the patients with hemorrhagic stroke. On CNS examination, gait was highly affected in stroke patients (n = 37, 74%), followed by speech (n = 33, 66%), cranial nerves (n = 23, 46%), and cerebellar signs (n = 5, 10%). However, none of the patients have found abnormalities in involuntary movements, meningeal signs, or the spine. With the examination of the motor system, it was found that patients with hypertonia (p = 0.016) and reduced power (p = 0.0080) were significantly higher among hemorrhagic strokes (p = 0.0080) than ischemic strokes. In the present study, the comorbidities such as diabetes mellitus and hypertension were almost equally distributed among hemorrhagic and ischemic stroke patients at 22 (44%) and 21 (42%), respectively, while smoking and alcohol were found to be 56% and 40%, respectively (Table 2).

**Table 2:** Comparison of the symptoms among the ischemic and hemorrhagic stroke patients The data is shown as frequency (N) and percentage (%); chi-square test *The p-value of <0.05 is considered significant; NS, non-significant DM, diabetes mellitus; HTN, hypertension; IHD, ischemic heart disease

Parameters	Ischemic stroke (n = 40), N (%)	Hemorrhagic stroke (n = 10), N (%)	Total, N (%)	Chi-square value	p-value
Common symptoms					
Facial palsy	17 (42.5%)	5 (50%)	22 (44%)	0.194	0.66^NS^
Speech disorder	26 (65%)	8 (80%)	34 (68%)	0.838	0.36^NS^
Hemiplegia	34 (85%)	7 (70%)	41 (82%)	1.269	0.26^NS^
Altered sensorium	20 (50%)	8 (80%)	28 (56%)	3.065	0.08^NS^
Seizure	10 (25.0%)	7 (70%)	17 (34%)	7.273	0.007*
Headache	2 (5%)	8 (80%)	10 (20%)	15.136	0.000*
Involuntary urination and defecation	7 (17.5%)	4 (40%)	11 (22%)	2.417	0.12^NS^
Vomiting	6 (15%)	4 (40%)	10 (20%)	3.283	0.07^NS^
CNS symptoms					
Gait	31 (75%)	6 (60%)	37 (74%)	1.323	0.25^NS^
Cranial nerves	19 (47.5%)	4 (50%)	23 (46%)	0.182	0.67^NS^
Speech	26 (65.0%)	7 (70.0%)	33 (66.0%)	0.093	0.76^NS^
Involuntary movements	0 (0%)	0 (0%)	0 (0%)		
Cerebellar signs	4 (10.0%)	1 (10.0%)	5 (10%)	0.001	1.000^NS^
Meningeal signs	0 (0%)	0 (0%)	0 (0%)		
Spine	0 (0%)	0 (0%)	0 (0%)		
Motor system	40 (100%)	10 (100%)	50 (100%)		
Tone					
Hypotonia	25 (62.5%)	2 (20.0%)	27 (54.0%)	9.21	0.01*
Normal	0 (0%)	0 (0%)	0 (0%)		
Hypertonia	15 (37.5%)	8 (80.0%)	23 (46.0%)		
Power					
Reduced (<3)	22 (57.5%)	10 (100%)	32 (64.0%)	7.033	0.008*
3-5	18 (42.5%)	0 (0%)	18 (36.0%)		
Reflex					
Reduced	22 (55.0%)	5 (50.0%)	27 (54.0%)	0.523	0.77^NS^
Normal	0 (0%)	0 (0%)	0 (0%)		
Exaggerated	18 (45.0%)	5 (50.0%)	23 (46.0%)		
Co-morbidities					
DM	17 (40%)	5 (50.0%)	22 (44%)	0.194	0.66^NS^
HTN	16 (37.5%)	5 (60.0%)	21 (42%)	0.34	0.56^NS^
IHD	3 (7.5%)	2 (20.0%)	05 (10%)	1.441	0.23^NS^
Smoking	25 (62.5%)	3 (30.0%)	28 (56%)	3.537	0.06^NS^
Alcohol	16 (40%)	4 (40.0%)	20 (40%)	0.001	1.000^NS^

The average fibrinogen level was much higher in patients with ischemic stroke compared to those with hemorrhagic stroke (441.5 ± 152.04 vs. 308.5 ± 107.3 mg/dL; p = 0.016). The mean prothrombin time was higher than normal among patients with ischemic stroke (15.92 ± 1.68 mg/dL) and hemorrhagic stroke (15.92 ± 1.68 mg/dL), but it was not significant. MRI of the brain revealed DW positive among all patients with ischemic stroke and GRE positive among all patients with hemorrhagic stroke (Table 3).

**Table 3:** Comparison of biochemical parameters among the ischemic and hemorrhagic stroke patients The data is shown as mean ± SD. Unpaired student’s t-test *The p-value of <0.05 is considered significant; NS, non-significant

Biochemical parameters	Ischemic stroke (n = 40), N (%)	Hemorrhagic stroke (n = 10), N (%)	Reference range	t-value	p-value
HB (gm/dL)	11.23 ± 1.95	11.86 ± 2.63	12-16	0.868	0.39^NS^
RBS (mg/dL)	146.05 ± 23.33	159.8 ± 33.06	110-140	1.541	0.13^NS^
Blood urea (mg/dL)	27.27 ± 5.03	25.1 ± 3.21	15-40	1.299	0.20^NS^
S. creatinine (mg/dL)	0.82 ± 0.15	0.85 ± 0.17	0.7-1.4	0.557	0.58^NS^
Total cholesterol (mg/dL)	207.57 ± 29.33	221.1 ± 30.35	140-200	1.299	0.20^NS^
S. fibrinogen (mg/dL)	441.5 ± 152.04	308.5 ± 107.3	200-400	2.682	0.01^*^
Prothrombin time (sec)	15.92 ± 1.68	15.9 ± 1.72	11-13.5	0.038	0.97^NS^
aPTT (sec)	33.07 ± 2.58	33.3 ± 3.19	25-40	0.268	0.79^NS^

The MRI findings showed that all 40 cases of ischemic stroke were DW positive, and all 10 cases of hemorrhagic stroke were GRE positive. MRA findings showed that in ischemic stroke patients, the majority of the cases had middle cerebral artery (MCA) involvement in nine (22.5%), followed by posterior cerebral artery (PCA) in four (10%), and internal carotid artery (ICA) and anterior cerebral artery (ACA) in two (5%) each. Overall, 15 (30%) of the patients were found normal. With regard to the site of involvement on MRA, in both ischemic and hemorrhagic stroke, 40% had right lobe involvement, and 32.5% and 40% had left lobe involvement (Table 4).

**Table 4:** MR angiography findings among the stroke patients

Parameters	Ischemic stroke (n = 40), N (%)	Hemorrhagic stroke (n = 10), N (%)
MR angiography finding		
Normal	5 (12.5%)	10 (100%)
Middle cerebral artery	9 (22.5%)	0 (0%)
Posterior cerebral artery	4 (10.0%)	0 (0%)
Internal carotid artery	2 (5.0%)	0 (0%)
Anterior cerebral artery	2 (5.0%)	0 (0%)
Vertebrobasilar artery	5 (12.5%)	0 (0%)
Multiple artery	13 (32.5%)	0 (0%)
MR angiography area finding		
Right lobe	16 (40%)	4 (40%)
Left lobe	13 (32.5%)	4 (40%)
Bilateral lobe	11 (27.5%)	2 (20%)

On MRI, in ischemic stroke, the majority of the patients had frontal and parietal involvement, which encompassed 37.5% each. In hemorrhagic stroke, the majority of the patients (40%) had parietal involvement, followed by the basal ganglia in 30% (Table 5).

**Table 5:** MRI findings among the ischemic and hemorrhagic stroke patients

MRI finding	Ischemic stroke (n = 40), N (%)	Hemorrhagic stroke (n = 10), N (%)
Frontal	15 (37.5%)	3 (30%)
Parietal	15 (37.5%)	1 (10%)
Temporal	8 (20%)	4 (40%)
Occipital	7 (17.5%)	1 (10%)
Midbrain	3 (12.5%)	0 (0%)
Pons	5 (12.5%)	1 (10%)
Medulla	3 (7.5%)	0 (0%)
Internal capsule	7 (17.5%)	2 (20%)
Basal ganglia	5 (12.5%)	3 (30%)
Cerebellum	4 (10%)	1 (10%)
Thalamus	5 (12.5%)	0 (0%)
White matter	5 (12.5%)	1 (10%)

## Discussion

Stroke continues to be a leading cause of death and disability worldwide, and its burden is increasing, particularly in developing countries like India. The findings of this study, conducted among 50 stroke patients, offer significant insights into the clinical presentation, biochemical profile, and MRI characteristics of cerebrovascular stroke, differentiating between ischemic and hemorrhagic subtypes. These insights help in guiding early diagnosis, therapeutic interventions, and prognostication.

Differentiating a stroke into an ischemic or hemorrhagic form is the initial stage in the diagnosis process, and it is crucial for subsequent stroke treatment. Since the prompt initiation of treatment determines the patient’s prognosis, the timing of the diagnosis is vital. Every stroke patient must have an MRI and MRA performed as a first line of investigation to rule out bleeding.

Our study demonstrated that ischemic stroke constituted 80% of cases, while hemorrhagic stroke comprised 20%. This proportion aligns closely with global and Indian epidemiological data, where ischemic strokes generally account for 80-85% of all strokes [2]. This predominance is explained by the higher prevalence of vascular risk factors such as atherosclerosis, hypertension, diabetes mellitus, and smoking, which mainly contribute to thrombotic and embolic occlusion of cerebral arteries, leading to ischemic events. The study by Kumar et al. [9] also reported a higher frequency of ischemic stroke (86.6%), reinforcing this trend in the Indian population. Likewise, in another study done by Patne and Chintale, the most prevalent kind of stroke was ischemic or cerebral infarction (68.29%), followed by hemorrhagic (31.69%) [10].

The age group of 51-80 years old had the highest incidence of stroke in this study (56%). A recent study conducted in India reveals a high prevalence of stroke in the age group >75 years when compared to the age group 45-59 (3.69 vs. 0.74) [11]. Likewise, in the study done by Kumar et al., the most common age group was 61-80 in 47% of the stroke patients [12]. Aging is associated with progressive endothelial dysfunction, arterial stiffness, and atherosclerotic plaque formation, all contributing to cerebrovascular events [13,14].

Our study showed a male preponderance (60%) with a male-to-female ratio of 1.5:1. Likewise, Itagi et al. revealed that 71% were males among the stroke patients [15]. This disparity may be attributed to differential exposure to risk factors like smoking and alcohol, as well as possible biological differences affecting vascular health [15].

The clinical profile revealed hemiplegia as the most common presenting symptom (82%), followed by speech disturbances (68%) and altered sensorium (56%). These results mirror previous findings that hemiplegia is the hallmark of focal neurological deficit in stroke due to disruption of corticospinal tracts [16]. Hemorrhagic stroke patients showed a significantly higher incidence of seizures (70%) and headache (80%) compared to ischemic stroke (25% and 5%, respectively), which concurs with the established understanding that cortical irritation from blood extravasation and raised intracranial pressure in hemorrhagic stroke precipitates such symptoms [17]. The increased incidence of hypertonia and reduced muscle power in hemorrhagic stroke compared to ischemic stroke also indicates a more severe neurological impairment, consistent with previous reports [10].

The distribution of neurological deficits, such as gait disturbance (74%), cranial nerve involvement (46%), and cerebellar signs (10%), underscores the variable vascular territories affected. Our cohort’s absence of involuntary movements, meningeal signs, or spinal involvement helped exclude other neurological pathologies, enhancing diagnostic specificity.

Hypertension and diabetes mellitus were present in approximately 42-44% of patients across both stroke subtypes, highlighting their pivotal role as modifiable risk factors. Smoking was significantly more prevalent among ischemic stroke patients (62.5%). Likewise, in a study done by Kotkunde and Kesarkar, 34.1% had hypertension, 9.3% had diabetes mellitus, and 14.2% had a smoking habit [18]. The link between smoking and stroke is multifactorial, including endothelial damage, increased platelet aggregation, and induction of prothrombotic states [19].

Raised fibrinogen levels in ischemic stroke patients compared to hemorrhagic stroke patients (441.5 ± 152.04 vs. 308.5 ± 107.3 mg/dL; p = 0.01) are noteworthy. In a study by Samir et al., the fibrinogen level was higher in ischemic stroke compared to the controls (538.31 ± 42.57 vs. 332.88 ± 52.18 mg/dL; p < 0.001) [20]. Fibrinogen, an acute-phase reactant, plays a central role in coagulation and thrombosis, and elevated levels have been associated with increased stroke severity and worse outcomes [21,22]. Elevated fibrinogen may reflect systemic inflammation and a prothrombotic milieu, promoting cerebral vessel occlusion. The role of fibrinogen as a predictor of ischemic stroke highlights its potential as a therapeutic target or prognostic marker. In another study by Khandait and Barai, the mean fibrinogen level was higher in ischemic stroke patients compared to the hemorrhagic stroke patients (584 vs. 552 mg/dL) [23]. Other biochemical parameters, such as hemoglobin, blood urea, serum creatinine, and cholesterol, did not show statistically significant differences. However, they remain essential for overall patient health and stroke risk stratification.

MRI remains the imaging modality of choice for acute stroke evaluation due to its superior sensitivity and specificity. In our study, all ischemic stroke patients were positive on DWI, which is known to detect ischemic lesions within minutes of symptom onset by identifying cytotoxic edema. This finding confirms the utility of DWI as a gold standard for early ischemic stroke diagnosis [24]. GRE sequences showed positivity in all hemorrhagic stroke patients, highlighting their sensitivity to hemorrhagic blood products and microbleeds.

MRA provided valuable vascular insights, with the MCA being the most frequently involved vessel (22.5%) in ischemic stroke, followed by the PCA (10%), the ICA (5%), and the ACA (5%). In a study done by Abdu et al., MCA is the largest cerebral artery and is most commonly affected in ischemic strokes in 49.2% of the cases [25]. Meanwhile, in our study, multiple artery involvement was seen in 32.5% of ischemic stroke cases, indicating widespread vascular disease.

The topographical MRI involvement in ischemic strokes predominantly affected the frontal and parietal lobes (37.5% each), regions supplied by the MCA. The parietal lobe (40%) and basal ganglia (30%) were predominant in hemorrhagic strokes. Likewise, in a study done by Abdu et al., the prevalence of infarctions in the basal ganglia and parietal lobe was 10.6% and 8.7%, respectively. Such anatomical correlations assist clinicians in confirming clinical diagnoses and prognosticating functional outcomes [25].

Limitations

Since this is a hospital-based research finding in a single setting, it could not be extrapolated to the broader population, and the temporality of association cannot be established in cross-sectional studies. In addition, a smaller sample size limits the statistical power and generalizability of our findings, and future multicentric longitudinal studies with larger cohorts are warranted to address these limitations.

## Conclusions

As people age, the prevalence of stroke rises. One of the leading causes of morbidity and death is acute ischemic stroke. Hypertension was a major risk factor for stroke. The most typical clinical manifestation was speech involvement after hemiplegia. The most common infarct site was the area supplied by the MCA. Thus, early identification of risk factors, abstinence from smoking, and management of these modifiable risk factors are essential in the prevention of stroke.
